# Supplementary material for: Who will benefit from bariatric surgery for diabetes? A protocol for an observational cohort study
Source: BMJ Open. 2021 Feb 10;11(2):e042355. doi: 10.1136/bmjopen-2020-042355 (PMC7878155; doi:10.1136/bmjopen-2020-042355)
Supplement: Author's manuscript [file bmjopen-2020-042355.draft_revisions.pdf]

BMJ Open is committed to open peer review. As part of this commitment we make the peer review history of every article we publish publicly available.

When an article is published we post the peer reviewers' comments and the authors' responses online. We also post the versions of the paper that were used during peer review. These are the versions that the peer review comments apply to.

The versions of the paper that follow are the versions that were submitted during the peer review process. They are not the versions of record or the final published versions. They should not be cited or distributed as the published version of this manuscript.

BMJ Open is an open access journal and the full, final, typeset and author-corrected version of record of the manuscript is available on our site with no access controls, subscription charges or pay-per-view fees (http://bmjopen.bmj.com).

If you have any questions on BMJ Open's open peer review process please email info.bmjopen@bmj.com

BMJ Open

Who will benefit from bariatric surgery for diabetes? A protocol for an observational cohort study

Journal:	*BMJ Open*
Manuscript ID	bmjopen-2020-042355
Article Type:	Protocol
Date Submitted by the Author:	02-Jul-2020
Complete List of Authors:	Kenkre, Julia; Imperial College London, Department of Metabolism, Digestion and Reproduction<br>Ahmed, Ahmed; Imperial College London, Department of Surgery and Cancer<br>purkayastha, sanjay; Imperial College London, Department of Surgery and Cancer<br>Malallah, Khalefah; Imperial College London, Department of Surgery and Cancer<br>Bloom, Stephen; Imperial College London, Clinical Director, North West London Pathology, Head of Division of Diabetes, Endocrinology and Metabolism, Imperial College, 6th Floor Commonwealth Building, Hammersmith Hospital, Imperial College London, Du Cane Road, London, W12 0NN<br>Blakemore, Alexandra; Brunel University College of Health and Life Sciences, Department of Life Sciences<br>Prevost, A; King's College London, Nightingale-Saunders Clinical Trials and Epidemiology Unit, King's Clinical Trials Unit<br>Tan, Tricia; Imperial College London, Department of Metabolism, Digestion and Reproduction
Keywords:	General diabetes < DIABETES & ENDOCRINOLOGY, Adult surgery < SURGERY, NUTRITION & DIETETICS

SCHOLARONE™
Manuscripts

I, the Submitting Author has the right to grant and does grant on behalf of all authors of the Work (as defined in the below author licence), an exclusive licence and/or a non-exclusive licence for contributions from authors who are: i) UK Crown employees; ii) where BMJ has agreed a CC-BY licence shall apply, and/or iii) in accordance with the terms applicable for US Federal Government officers or employees acting as part of their official duties; on a worldwide, perpetual, irrevocable, royalty-free basis to BMJ Publishing Group Ltd ("BMJ") its licensees and where the relevant Journal is co-owned by BMJ to the co-owners of the Journal, to publish the Work in this journal and any other BMJ products and to exploit all rights, as set out in our licence.

The Submitting Author accepts and understands that any supply made under these terms is made by BMJ to the Submitting Author unless you are acting as an employee on behalf of your employer or a postgraduate student of an affiliated institution which is paying any applicable article publishing charge ("APC") for Open Access articles. Where the Submitting Author wishes to make the Work available on an Open Access basis (and intends to pay the relevant APC), the terms of reuse of such Open Access shall be governed by a Creative Commons licence – details of these licences and which Creative Commons licence will apply to this Work are set out in our licence referred to above.

Other than as permitted in any relevant BMJ Author's Self Archiving Policies, I confirm this Work has not been accepted for publication elsewhere, is not being considered for publication elsewhere and does not duplicate material already published. I confirm all authors consent to publication of this Work and authorise the granting of this licence.

For peer review only

TITLE PAGE

TITLE: Who will benefit from bariatric surgery for diabetes? A protocol for an observational cohort study.

Authors:

Dr Julia Kenkre, [1]

Mr Ahmed Ahmed[4]

Mr Sanjay Purkayastha[4]

Dr Khalefah Malallah [4]

Prof Stephen Bloom, [1]

Prof Alexandra Blakemore, [2]

Prof A. Toby Prevost, [3]

Prof Tricia Tan[1]

Author affiliations

1. Section of Endocrinology and Investigative Medicine, Imperial College, London. UK
2. Department of Life Sciences, Brunel University, London. UK
3. Nightingale-Saunders Clinical Trials and Epidemiology Unit, King's Clinical Trials Unit, King's College, London. UK
4. Department of Surgery and Cancer, Imperial College, London. UK

For peer review only

For peer review only - http://bmjopen.bmj.com/site/about/guidelines.xhtml

Corresponding author Tricia Tan, Section of Endocrinology and Investigative Medicine, 6th Floor Commonwealth Building, Imperial College Hammersmith Hospital Campus, Du Cane Road, London, W12 0NN. t.tan@imperial.ac.uk

WORD COUNT 2869

For peer review only

ABSTRACT

Introduction Type 2 diabetes mellitus (T2DM) and obesity are pandemic diseases that lead to a great deal of morbidity and mortality. The most effective treatment for obesity and T2DM is bariatric surgery; it can lead to long-term diabetes remission with 4 in 10 of those undergoing surgery having normal blood glucose on no medication one year post-operatively. However, surgery carries risks and, additionally, due to resource-limitation, there is a restricted number of patients who can access this treatment. Moreover, not all those who undertake surgery respond equally well metabolically. The objective of the current research is to prospectively investigate predictors of T2DM response following bariatric surgery, including those directly involved in its aetiopathogenesis such as fat distribution and genetic variants. This will inform development of a clinically applicable model to help prioritise this therapy to those predicted to have remission.

Methods and analysis A prospective multicentre observational cohort study of adult patients with T2DM and obesity undergoing Roux-en-Y gastric bypass (RYGB) surgery. Patients will be comprehensively assessed before surgery to determine their clinical, metabolic, psychological, genetic and fat distribution profiles. A multivariate logistic regression model will be used to assess the value of the factors derived from the pre-operative assessment in terms of prediction of diabetes remission.

Ethics and dissemination. Formal ethics review was undertaken with a favourable opinion (UK HRA RES reference number 18/LO/0931). The dissemination plan is to

For peer review only

present the results at conferences, in peer-reviewed journals as well as lay media and to patient organisations.

Trial registration number:

ClinicalTrials.gov Identifier: NCT03842475

ARTICLE SUMMARY

Strengths and limitations of this study

- One of the first studies to prospectively and comprehensively profile patients with type 2 diabetes undergoing bariatric surgery to assess predictors of diabetes remission.
- Collects prospective data on factors known to be involved in the aetiopathogenesis of T2DM such as genetics and fat distribution.
- A biorepository of tissue samples will be collected to allow multi-omics analysis.
- Long-term follow up for up to ten years will enable assessment of the durability of remission.
- The primary limitation is that this is a preliminary study; it will identify and internally validate predictors and will require further external validation in other cohorts.

KEY WORDS

Obesity, Bariatric Surgery, Diabetes Mellitus, Type 2

For peer review only

INTRODUCTION

Currently the majority of adults globally are obese or overweight. The increasing number of those with obesity is closely mirrored by the increasing prevalence of diabetes; currently over 400 million adults are estimated to be affected, 90% of whom have T2DM. (1) The close relationship between obesity, particularly 'central' or abdominal obesity, and T2DM has now been shown in multiple studies. (2, 3) Obesity, as defined by ethnicity-specific body mass index (BMI) cut-offs, is the principal modifiable risk factor for developing T2DM. However, the weight threshold at which T2DM develops varies between individuals, with significant variation amongst different ethnicities exemplifying two of the important factors that are thought to influence risk: individual pattern of where fat is deposited and underlying genetic predisposition.(4-9)

Epidemiological studies have shown those with a higher waist: hip ratio (WHR), representing centrally-deposited fat, are more likely to develop the disease.(3) Central fat or higher intra-abdominal visceral fat deposition is known to be metabolically more damaging with increased likelihood of developing abnormalities in glycaemia, blood pressure and lipid profiles.(10) However, it is not simply central adiposity but also ectopic fat that may have pathophysiological consequences.(11) Evidence is accumulating regarding the detrimental effect on glycaemia from fat ectopically deposited within liver, skeletal muscle and pancreas.(12)

T2DM shows heritability and many pathogenic variants have been discovered which influence β cell functioning and insulin resistance.(13, 14) Moreover, genetic susceptibility to T2DM is known to be correlated with phenotypic changes in fat distribution: genetic variants associated with insulin resistance have been found to be correlated with lower subcutaneous adipose tissue (SAT) and a genetic predisposition

For peer review only

to diabetes has been found in those with impaired SAT adipogenesis and increased waist circumference.(15) Combining GWAS data to derive personalised genetic risk, whilst challenging, has been successfully achieved in other conditions with the potential to add further precision to a predictive score. (16, 17)

Remission of diabetes

The most effective treatment for obesity and diabetes is bariatric surgery; it has been relabelled as 'metabolic surgery' to highlight its primary utility as a therapy for diabetes and other related metabolic dysfunctions, including dyslipidaemia and non-alcoholic fatty liver disease, as opposed to primarily treating excess weight.(18) Randomised control trials have shown bariatric surgery consistently out performs best-medical therapy in improving glycaemic control and leads to durable remission in a significant proportion.(19, 20) RYGB, the gold-standard procedure for those with diabetes, involves anastomosis of a small pouch of stomach to jejunum such that food is diverted to the jejunum, bypassing the majority of the stomach and the duodenum. Following RYGB, remission of diabetes (as defined by markers of normoglycaemia in the absence of anti-diabetic medication) occurs in 4 in 10 patients at one year and this remission is long-lasting: the majority of these patients remain in remission at 5 years.(18-20) Furthermore, surgery also leads to a reduction in micro- and macrovascular complications associated with diabetes.(21)

No single unifying mechanism found thus far can explain the improvement in glycaemic control seen in those with T2DM following RYGB. Whilst it is clear that the 25-30% weight loss seen by 12-18 months post-operatively plays a significant role, improvement in glycaemia occurs within days following surgery, prior to any significant weight reduction, suggesting a role for weight-loss independent mechanisms.(22)

For peer review only

Reduced calorie intake post-surgery is one such putative weight-loss independent mechanism for the immediate improvement in glycaemia as those on a very low-calorie diet can achieve reversal of the metabolic features of diabetes.(23) However, caloric restriction normally also leads to homeostatic compensation or counter-regulation including increased appetite, reduced energy expenditure and secretion of orexigenic gut hormones. In contrast, following surgery, appetite is known to reduce and weight-adjusted resting energy expenditure may increase. (24-26)

It is likely that caloric restriction and weight loss are only two of many mechanisms that contribute to the post-operative metabolic improvement seen. Insulinotropic and anorexigenic gut hormones including glucagon like peptide 1 (GLP-1), Peptide YY (PYY) and oxyntomodulin increase following surgery.(27, 28) PYY and GLP-1 have been implicated in the restoration of insulin secretion after RYGB.(29, 30) Furthermore, infusion of such hormones leads to improved metabolic control.(31) Changes in their secretion have been correlated with post-operative metabolic and weight loss response.(30, 32, 33) Associated with these findings are the increased levels of plasma bile acids after surgery which have been correlated with increased levels of GLP-1.(34) Bile acids have been implicated in the improvement in glycaemia via signalling through the nuclear farnesoid X receptor (FXR) and G-protein-coupled TGR5 receptors.(35, 36) TGR5 activation leads to increased secretion of insulinotropic GLP-1 and modulates insulin sensitivity in tissues such as muscle and brown adipose tissue.(37) Whilst changes in bile acids play a role in metabolic improvement, this still remains an area of active research.

Further adding to the complexity of the mechanism of remission, differences in the gut microbiota i.e. the microorganisms commensal within the gut, have been seen between

For peer review only

lean and obese individuals.(38) There is some evidence that, as a regulator of the host's metabolism, the gut microbiota may be associated with obesity through several mechanisms such as enhancement of fat storage.(39) Significant changes occur in the gut microbiota following surgery (40) but the causal relationship between the microbiotal changes and metabolic improvements, i.e. whether the metabolic improvements come from the microbiotal changes or vice versa, has yet to be established. Furthermore, there is a bi-directional relationship between the microbiome and bile acids: whilst the gut microbiota have a role in converting primary to secondary bile acids, bile acids can affect the diversity of gut microbiota.(41)

We conclude that to explore the mechanism of remission further a comprehensive multi-omics approach must therefore be employed.

Predicting diabetes remission following surgery

There is a substantial disparity between the numbers eligible for bariatric surgery and the resources available to deliver it; in the UK two million people fulfil the criteria but surgery is only being performed in c. 5600 patients per year.(42) Furthermore, there is variation in the metabolic response between individuals such that less than half are expected to have complete diabetes remission at one year following surgery. If we can better predict who will benefit from bariatric surgery by remission of diabetes we can better inform patients and direct the surgery to where it will be most effective.(43) Currently in the UK, BMI cut-offs dictate eligibility for surgery, along with more recently introduced diabetes duration and ethnicity criteria.(44) However, baseline BMI does not correlate well with the likelihood of diabetes remission thus other predictors are necessary to better inform eligibility based on intended benefit.(45)

For peer review only

Predictive factors have been found in multiple, mainly retrospective studies and some have been incorporated into models of diabetes remission.(46-48) These models have tried to predict diabetes remission based on easily available clinical data making them translatable to everyday practice. However, they remain limited in their clinical applicability.(49) Moreover, none have incorporated fat distribution or genetic risk factors which have important aetiopathological roles in T2DM. Furthermore, they are, in most cases, based on retrospective studies. One problem faced in this instance is that the consensus definition of diabetes remission depends on withdrawal of pharmacological therapies for T2DM for one year. There is often no standardised pathway to assess the remission status after surgery, which can lead to differing practice amongst clinicians thus hampering the ability to define remission status with accuracy.

This study will use genetic profiling, identifying genetic variants associated with fat distribution patterns, β-cell dysfunction, T2DM or insulin resistance, combined into an individual genetic-risk score for each patient. (50, 51) Moreover, it will assess the distribution pattern of total body fat in addition to ectopic fat deposition within the liver, muscle and pancreas. These variables will be tested to assess if they are predictive of diabetes remission. In addition, patients will be profiled for variation in biomarkers postulated to be mechanistically involved in diabetes remission including gut hormones, microbiotal changes and bile acids.

OBJECTIVES

Primary objective

To develop and internally validate a predictive model of diabetes remission following bariatric surgery.

For peer review only

Secondary objectives

To assess changes in diabetes and obesity-related co-morbidities following surgery and investigate mechanism of diabetes remission.

METHODS AND ANALYSIS

This is a prospective, multicentre observational cohort study investigating predictors of diabetes remission in patients with diabetes/prediabetes undergoing bariatric surgery.

Participants and selection criteria

**Inclusion criteria**	Patients with prediabetes or diabetes eligible for bariatric surgery on the NHS, i.e. BMI ≥35 with a co-morbidity or ≥40, lower BMIs accepted if of Asian origin or recent-onset diabetes, referred to Imperial Weight Centre or Chelsea and Westminster NHS Trust(44, 52)
	Stable weight for at least 3 months
**Exclusion criteria**	Current pregnancy
	Inability to give informed consent
	Type 1 diabetes, low fasting C-peptide, secondary diabetes or absence of β-cell function
	Unable to undergo DEXA
	Cirrhosis, ascites, or other condition that may modify body fat composition e.g. underlying malignancy
	Participation in a concomitant interventional trial within the last 3 months that would affect the current study results or

For peer review only

	mean that undergoing the study would be too burdensome for the participant.
	Unable to understand English.

Table 1. Inclusion and exclusion criteria

Outcomes

The primary outcome is diabetes remission (partial and complete) or diabetes remission (complete) measured post-operatively at 15 months. This will be defined by American Diabetes Association criteria, such that complete remission is fasting glucose <5.6 mmol/L, HbA1c <42 mmol/mol on no anti-diabetic agents, and partial remission is defined as fasting glucose 5.6-6.9 mmol/L, HbA1c <48 mmol/mol on no anti-diabetic agents. Patients will be seen at 3 months as part of routine clinical follow-up and will have anti-diabetic medication stopped according to a standardised pathway. An assessment of diabetes remission will also be made at 1, 2, 3 and 5 years.

In addition to the primary outcome we will collect phenotyping data to allow assessment of diabetes severity and obesity associated co-morbidities.

Exploratory outcomes:

• Change in epigenetic data including DNA methylation, histone modifications and non-coding RNA in blood, liver, muscle, fat (subcutaneous and visceral) and stomach and small bowel and spermatozoa

• Changes in the transcriptome in blood, liver, muscle, fat (subcutaneous and visceral) and stomach and small bowel and spermatozoa

For peer review only

• Changes in gut hormone profiles in response to a mixed meal

• Histological scoring of specimens for NAFLD/NASH

• Changes in gut microbiotal diversity, relative abundance of selected phyla (e.g. Bacteroides and Firmicutes), and frequency of operational taxonomic units.

Sample size calculation

Using a logistic regression model to predict a 75% partial and completed diabetes remission rate from multiple predictors, a sample size of 150 provides 90% power at the two-sided 5% significance level to detect an odds ratio of 2.0 per standard deviation of any continuous predictor. This allows for up to a 0.4 correlation between a predictor and the others in the model, and 7.5% loss to follow-up. The area under ROC curve, overall performance measure, will be estimated with a 95% confidence interval width of at most +/-0.1 for an area above 0.65.

Measures

Measures are summarised in Table 2.

Visit number	1	Surgery	2	3	4	5
**Month**	**Pre-op** *Co-variates measured here*	**0**	**15** *Primary outcome measured here*	**24**	**36**	**60**
**Anthropometrics* and bioimpedance measurement**	✓	Weight only	✓	✓	✓	✓

For peer review only

**Blood tests including Hb1c and fasting glucose to enable assessment of remission +**	✓	✓	✓	✓	✓	✓
**Mixed meal test (MMT)**	✓		✓			
**Psychological and exercise screening questionnaires**	✓		✓	✓	✓	✓
**Food intake assessed by a 4-7-day food diary**	✓		✓			✓
**24-hour blood pressure and capillary or continuous glucose monitoring**	✓		✓			
**Urine and stool sample**	✓	✓	✓			✓
**Fat biopsy**		✓	✓			
**Liver, muscle and gut biopsy**		✓				
**Whole body DEXA**	✓		✓			
**Sperm sample (Male participants only)**	✓		✓			
**Whole body MRI**	✓		✓			
**Endoscopy and colonoscopy + biopsy**	✓		✓			

For peer review only - http://bmjopen.bmj.com/site/about/guidelines.xhtml

**(Selected participants only)**						

Table 2. Time and Events table of measures that participants will undergo throughout the study. *Anthropometrics includes height, waist, hip and neck circumference. + Blood tests include glucose, insulin, C-peptide, gut hormones, free fatty acids, bile acids, plasma metabolomics, DNA, RNA, cortisol, HbA1c, lipids and FGF 19/21

In addition to collection of a full clinical history the following measures will be undertaken:

Mixed Meal test

Pre-operatively and at 15 months following their first study visit participants will undergo a mixed meal test, using a stimulus of 14 g protein, 12.9 g fat, 39.6 g carbohydrate, 330 kcal, 137.5 ml (Ensure Compact, Abbott). Prior to this, withdrawal of diabetes medications which will occur using a standardised pathway. Participants will have blood samples taken to measure HbA1c, fasting and stimulated insulin/ C-peptide/ glucose, gut hormones, free fatty acids, bile acids, plasma metabolites and FGF-19/-21.

Questionnaires

The following questionnaires will be used to examine psychological wellbeing: Alcohol Use Disorders Identification Test (AUDIT), Eating Disorder Examination Questionnaire (EDEQ), Difficulty in Emotion Regulation Scale (DERS), Hospital Anxiety and Depression Scale (HADS) and Short Form 36 (SF-36). The Temperament and Character Inventory (TCI) will also be collected at baseline alone. The International Physical Activity Questionnaire (IPAQ) short form will be used to assess exercise levels.

For peer review only

Questionnaires will be analysed to assess if they impact on likelihood of obtaining remission and, in subsequent visits if they modify the consequences of surgery i.e. maintenance of diabetes remission. The TCI will be used to assess whether personality subtype predicts metabolic response to surgery.

Metabolic status

Lipid profile along with 24-hour blood pressure monitoring will be analysed to further assess the response of these cardiometabolic risk factors to surgery. Continuous glucose measurement sensors or capillary glucose readings will be undertaken to give an alternative measure of glycaemic control.

Body composition

All patients will undergo a whole body DEXA prior to and 15 months following surgery. This is a validated tool to estimate visceral adipose tissue.(53, 54)

Where feasible, participants will undergo a gold standard assessment of visceral adipose tissue using MRI which will also quantify liver, muscle and pancreatic fat.

DNA: samples will be collected and analysed using a SNP array to assess presence of genetic variants related to T2DM and fat patterning. A weighted genetic risk score will be calculated individually.

Omics

These will be used for to explore possible mechanisms explaining the metabolic changes before and after surgery.

For peer review only

Transcriptomics/epigenomics/proteomics: Longitudinal tissue samples will be collected where possible and will undergo multi-omics assessment.

Microbiotal analysis: Longitudinal samples of stool will be collected to assess the microbiome.

Metabolomics: Longitudinal fasting urine and plasma metabolomic samples will be collected pre and following surgery

Predictors

Candidate predictors (for examples, see Table 3) have been selected based on those identified in previous retrospective cohort studies. Two unique predictors, fat distribution and genetic risk score, will also be tested. We will then cautiously extend the model to address other factors in a univariate manner.

Predictor	Units/categories	Variable type	Source data	Previous studies
**Age**	years	Continuous	Clinical history	(46, 47, 55, 56)
**BMI or Weight**	kg/m^2 or kg	Continuous	V1	(57, 58)
**C peptide**	pmol/L	Continuous	Mixed meal test	(46, 55, 59)
**Diabetes duration**	years	Continuous	Clinical history	(46, 55, 56, 60-64)
**Fasting plasma**	mmol/L	Continuous	Blood test	(65)

For peer review only

**glucose**				
**Fat distribution**	Visceral fat and Android: Gynoid fat	Continuous	(DEXA, impedance, MRI)	
**Genetic risk score**	Weighted genetic risk score	Categorical	DNA V1	
**HbA1c with medications**	mmol/mol<br><br>Insulin, sulphonylureas, insulin sensitising agents, GLP-1 analogues, DPP-IV inhibitors, SGLT-2 inhibitors	Continuous: will interact with medications since directly affected by them	Blood test	(47, 56, 57, 61-64, 66-68)
**HOMA 2**	Using interactive 24-variable assessment of homeostatic model		Mixed meal test	(69)
**Hypertension**	Present/Absent<br><br>Controlled by 1-5 agents	Categorical	Medical history, 24-hour blood pressure monitoring	
**Insulinogenic**	δ insulin (0-30 min)	Continuous	Mixed meal	

For peer review only

**index**	/ δ glucose (0-30 min)		test	
**Presence of fatty liver disease as assessed by non-invasive markers**	NAFLD liver fat score, Fatty Liver Index, FIB-4	Categorical	Medical history, MRI, Non-invasive markers including liver function tests	(70)
**Presence of diabetes complications**	Microvascular disease (including subgroups)<br><br>Macrovascular disease<br><br>(including subgroups)	Categorical	Clinical history	(55)
**Presence of ectopic fat**	% liver fat<br><br>% muscle fat<br><br>% pancreatic fat	Continuous	MRI	(71)
**Sex**	Male/Female	Categorical	Clinical history	
**TCI-R scoring**	Novelty seeking,	Continuous	V1	

	Harm avoidance, Reward Dependence, Persistence, Self-Directedness, Cooperativeness and Self-Transcendence			
**Waist: hip ratio**	Measured in cm	Continuous	V1	

Table 3. Candidate predictors of diabetes remission. V1 is study visit 1.

A logistic regression model predicting diabetes remission assessed at the 15-months visit will be built using the prospective cohort data. This was chosen in preference to an ordinal regression approach, such as the proportional odds model, because of the need to have a clear and independent interpretation of the predictive contribution both to complete remission and to complete and partial remission. Logistic regression was preferred over another alternative approach, discriminant analysis, for the same reason and because logistic regression provides predicted probabilities for individual patients which can then be then used within ROC curve analysis to provide estimates of sensitivity and specificity of the regression models measuring their accuracy to predict the diabetes remission outcome of bariatric surgery.

Initially, the effect of each of the clinical predictors and other covariates (patient demographic factors) of interest on each outcome is to be fitted in univariable models. Any clinical predictor that is significant at a threshold of p-value ≤0.20 will then be included in a multivariable model predicting the outcome of diabetes remission. Clinical

For peer review only

predictors and other covariates will then be eliminated in stepwise fashion until each remaining predictor is significant at the p-value=0.05 threshold and the models are found to be stable. Pattern of fat storage will be measured by VAT mass, SAT mass and VAT/SAT ratio, and genes influencing diabetes risk and fat deposition will be measured by the Genetic Risk Score using the approach of Fava et al. (51) These will be added to the model, if not already included, to allow their association with diabetes remission to be formally quantified and tested. We will cautiously extend the model to address other factors in a univariate manor. Internal validation of the selection of the predictive model will be done using bootstrap sampling with re-application of the model selection procedure using at least 1000 replications. The performance of the prediction model under different scenarios will be assessed, primarily by obtaining fitted probabilities of remission from the model to estimate the area under the ROC curve, and also by demonstrating the sensitivity, specificity, positive predictive value and negative predictive value across a series of cut points representing a range of scenarios including high sensitivity, high specificity and equivalent sensitivity and specificity.

Survival curves will be plotted to show the proportion remaining free from diabetes over the longer term according to categories of predictors identified to be important.

Patient and public involvement

Patients were consulted about the study objectives and selected study procedures to assess acceptability.

ETHICS AND DISSEMINATION:

For peer review only

This study complies with the Declaration of Helsinki and the principles of Good Clinical Practice. The researchers plan to disseminate results at conferences, in peer-reviewed journals as well as lay media and to patient organisations.

DATA STATEMENT

Anonymized data from the study will be made available upon request from the Principal Investigator upon reasonable request. ClinicalTrials.gov Identifier: NCT03842475

REFERENCES

1. Federation ID. IDF DIABETES ATLAS, Eight edition. 2017.

2. Neeland IJ, Turer AT, Ayers CR, et al. Dysfunctional adiposity and the risk of prediabetes and type 2 diabetes in obese adults. JAMA : the journal of the American Medical Association. 2012;308(11):1150-9.

3. The InterAct C. Long-Term Risk of Incident Type 2 Diabetes and Measures of Overall and Regional Obesity: The EPIC-InterAct Case-Cohort Study. PLoS Med. 2012;9(6):e1001230.

4. Taylor R. Pathogenesis of type 2 diabetes: tracing the reverse route from cure to cause. Diabetologia. 2008;51(10):1781-9.

5. Heymsfield SB, Peterson CM, Thomas DM, Heo M, Schuna JM, Jr. Why are there race/ethnic differences in adult body mass index-adiposity relationships? A quantitative critical review. Obesity reviews : an official journal of the International Association for the Study of Obesity. 2016;17(3):262-75.

6. Taylor R, Holman RR. Normal weight individuals who develop type 2 diabetes: the personal fat threshold. Clin Sci (Lond). 2015;128(7):405-10.

7. Wildman RP, Muntner P, Reynolds K, McGinn AP, Rajpathak S, Wylie-Rosett J, et al. The Obese Without Cardiometabolic Risk Factor Clustering and the Normal Weight With Cardiometabolic

For peer review only

Risk Factor Clustering: Prevalence and Correlates of 2 Phenotypes Among the US Population (NHANES 1999-2004). JAMA internal medicine. 2008;168(15):1617-24.

8. Bouchard C, Tremblay A, Despres JP, Nadeau A, Lupien PJ, Theriault G, et al. The response to long-term overfeeding in identical twins. The New England journal of medicine. 1990;322(21):1477-82.

9. Shai I, Jiang R, Manson JE, Stampfer MJ, Willett WC, Colditz GA, et al. Ethnicity, Obesity, and Risk of Type 2 Diabetes in Women. A 20-year follow-up study. 2006;29(7):1585-90.

10. Despres JP, Lemieux I. Abdominal obesity and metabolic syndrome. Nature. 2006;444(7121):881-7.

11. Ferrara D, Montecucco F, Dallegri F, Carbone F. Impact of different ectopic fat depots on cardiovascular and metabolic diseases. Journal of cellular physiology. 2019.

12. Jaghutriz B, Wagner R, Machann J, Stefan N, Peter A, Siegel-Axel DI, et al. Association of Pancreas Fat with Impaired Insulin Secretion Depends on Liver Fat and Circulating Fatty Acids. Diabetes. 2018;67(Supplement 1):1819-P.

13. Langenberg C, Lotta LA. Genomic insights into the causes of type 2 diabetes. The Lancet. 2018;391(10138):2463-74.

14. Fuchsberger C, Flannick J, Teslovich TM, Mahajan A, Agarwala V, Gaulton KJ, et al. The genetic architecture of type 2 diabetes. Nature. 2016;536(7614):41-7.

15. Shungin D, Winkler TW, Croteau-Chonka DC, Ferreira T, Locke AE, Mägi R, et al. New genetic loci link adipose and insulin biology to body fat distribution. Nature. 2015;518(7538):187-96.

16. Pedersen HK, Gudmundsdottir V, Pedersen MK, Brorsson C, Brunak S, Gupta R. Ranking factors involved in diabetes remission after bariatric surgery using machine-learning integrating clinical and genomic biomarkers. Npj Genomic Medicine. 2016;1:16035.

17. Khera AV, Chaffin M, Aragam KG, Haas ME, Roselli C, Choi SH, et al. Genome-wide polygenic scores for common diseases identify individuals with risk equivalent to monogenic mutations. Nature genetics. 2018;50(9):1219-24.

For peer review only

18. Schauer PR, Bhatt DL, Kirwan JP, Wolski K, Aminian A, Brethauer SA, et al. Bariatric Surgery versus Intensive Medical Therapy for Diabetes — 5-Year Outcomes. New England Journal of Medicine. 2017;376(7):641-51.

19. Schauer PR, Kashyap SR, Wolski K, Brethauer SA, Kirwan JP, Pothier CE, et al. Bariatric Surgery versus Intensive Medical Therapy in Obese Patients with Diabetes. New England Journal of Medicine. 2012;366(17):1567-76.

20. Mingrone G, Panunzi S, De Gaetano A, Guidone C, Iaconelli A, Leccesi L, et al. Bariatric surgery versus conventional medical therapy for type 2 diabetes. The New England journal of medicine. 2012;366(17):1577-85.

21. Sjostrom L, Peltonen M, Jacobson P, Ahlin S, Andersson-Assarsson J, Anveden A, et al. Association of bariatric surgery with long-term remission of type 2 diabetes and with microvascular and macrovascular complications. JAMA : the journal of the American Medical Association. 2014;311(22):2297-304.

22. Courcoulas AP, Christian NJ, Belle SH, Berk PD, Flum DR, Garcia L, et al. Weight change and health outcomes at 3 years after bariatric surgery among individuals with severe obesity. JAMA : the journal of the American Medical Association. 2013;310(22):2416-25.

23. Lim EL, Hollingsworth KG, Aribisala BS, Chen MJ, Mathers JC, Taylor R. Reversal of type 2 diabetes: normalisation of beta cell function in association with decreased pancreas and liver triacylglycerol. Diabetologia. 2011;54(10):2506-14.

24. Al-Najim W, Docherty NG, le Roux CW. Food Intake and Eating Behavior After Bariatric Surgery. Physiol Rev. 2018;98(3):1113-41.

25. Faria SL, Faria OP, Buffington C, de Almeida Cardeal M, Rodrigues de Gouvea H. Energy expenditure before and after Roux-en-Y gastric bypass. Obesity surgery. 2012;22(9):1450-5.

26. Wilms B, Ernst B, Thurnheer M, Schmid SM, Spengler CM, Schultes B. Resting energy expenditure after Roux-en Y gastric bypass surgery. Surgery for obesity and related diseases : official journal of the American Society for Bariatric Surgery. 2018;14(2):191-9.

For peer review only

27. Laferrère B. Diabetes remission after bariatric surgery: is it just the incretins? International journal of obesity (2005). 2011;35 Suppl 3(0 3):S22-S5.

28. Dirksen C, Jorgensen NB, Bojsen-Moller KN, Jacobsen SH, Hansen DL, Worm D, et al. Mechanisms of improved glycaemic control after Roux-en-Y gastric bypass. Diabetologia. 2012;55(7):1890-901.

29. Ramracheya Reshma D, McCulloch Laura J, Clark A, Wiggins D, Johannessen H, Olsen Magnus K, et al. PYY-Dependent Restoration of Impaired Insulin and Glucagon Secretion in Type 2 Diabetes following Roux-En-Y Gastric Bypass Surgery. Cell Reports.15(5):944-50.

30. Nannipieri M, Baldi S, Mari A, Colligiani D, Guarino D, Camastra S, et al. Roux-en-Y Gastric Bypass and Sleeve Gastrectomy: Mechanisms of Diabetes Remission and Role of Gut Hormones. The Journal of Clinical Endocrinology & Metabolism. 2013;98(11):4391-9.

31. Torekov SS, Kipnes MS, Harley RE, Holst JJ, Ehlers MR. Dose response of subcutaneous GLP-1 infusion in patients with type 2 diabetes. Diabetes, obesity & metabolism. 2011;13(7):639-43.

32. le Roux CW, Welbourn R, Werling M, Osborne A, Kokkinos A, Laurenius A, et al. Gut hormones as mediators of appetite and weight loss after Roux-en-Y gastric bypass. Annals of surgery. 2007;246(5):780-5.

33. Nielsen MS, Ritz C, Wewer Albrechtsen NJ, Holst JJ, le Roux CW, Sjödin A. Oxyntomodulin and glicentin may predict the effect of bariatric surgery on food preferences and weight loss. The Journal of Clinical Endocrinology & Metabolism. 2020.

34. Kaska L, Sledzinski T, Chomiczewska A, Dettlaff-Pokora A, Swierczynski J. Improved glucose metabolism following bariatric surgery is associated with increased circulating bile acid concentrations and remodeling of the gut microbiome. World journal of gastroenterology. 2016;22(39):8698-719.

35. Patti ME, Houten SM, Bianco AC, Bernier R, Larsen PR, Holst JJ, et al. Serum bile acids are higher in humans with prior gastric bypass: potential contribution to improved glucose and lipid metabolism. Obesity (Silver Spring). 2009;17(9):1671-7.

For peer review only

36. Gerhard GS, Styer AM, Wood GC, Roesch SL, Petrick AT, Gabrielsen J, et al. A role for fibroblast growth factor 19 and bile acids in diabetes remission after Roux-en-Y gastric bypass. Diabetes care. 2013;36(7):1859-64.

37. Wang W, Cheng Z, Wang Y, Dai Y, Zhang X, Hu S. Role of Bile Acids in Bariatric Surgery. Front Physiol. 2019;10:374.

38. Turnbaugh PJ, Hamady M, Yatsunenko T, Cantarel BL, Duncan A, Ley RE, et al. A core gut microbiome in obese and lean twins. Nature. 2009;457(7228):480-4.

39. Magouliotis DE, Tasiopoulou VS, Sioka E, Chatedaki C, Zacharoulis D. Impact of Bariatric Surgery on Metabolic and Gut Microbiota Profile: a Systematic Review and Meta-analysis. Obesity surgery. 2017.

40. Li JV, Ashrafian H, Bueter M, Kinross J, Sands C, le Roux CW, et al. Metabolic surgery profoundly influences gut microbial-host metabolic cross-talk. Gut. 2011;60(9):1214-23.

41. Cani PD, Van Hul M, Lefort C, Depommier C, Rastelli M, Everard A. Microbial regulation of organismal energy homeostasis. Nature Metabolism. 2019;1(1):34-46.

42. Society BOaMS. Clinical Outcomes 2017-18. 2018.

43. Ahmad A, Laverty AA, Aasheim E, Majeed A, Millett C, Saxena S. Eligibility for bariatric surgery among adults in England: analysis of a national cross-sectional survey. JRSM Open. 2014;5(1).

44. NICE. Obesity: identification, assessment and management of overweight and obesity in children, young people and adult. 2014.

45. Panunzi S, De Gaetano A, Carnicelli A, Mingrone G. Predictors of remission of diabetes mellitus in severely obese individuals undergoing bariatric surgery: do BMI or procedure choice matter? A meta-analysis. Annals of surgery. 2015;261(3):459-67.

46. Lee WJ, Hur KY, Lakadawala M, Kasama K, Wong SK, Chen SC, et al. Predicting success of metabolic surgery: age, body mass index, C-peptide, and duration score. Surgery for obesity and related diseases : official journal of the American Society for Bariatric Surgery. 2013;9(3):379-84.

For peer review only

47. Still CD, Wood GC, Benotti P, Petrick AT, Gabrielsen J, Strodel WE, et al. Preoperative prediction of type 2 diabetes remission after Roux-en-Y gastric bypass surgery: a retrospective cohort study. The lancet Diabetes & endocrinology. 2014;2(1):38-45.

48. Zhang R, Borisenko O, Telegina I, Hargreaves J, Ahmed AR, Sanchez Santos R, et al. Systematic review of risk prediction models for diabetes after bariatric surgery. BJS. 2016;103(11):1420-7.

49. Tharakan G, Scott R, Szepietowski O, Miras AD, Blakemore AI, Purkayastha S, et al. Limitations of the DiaRem Score in Predicting Remission of Diabetes Following Roux-En-Y Gastric Bypass (RYGB) in an ethnically Diverse Population from a Single Institution in the UK. Obesity surgery. 2016.

50. Blakemore AIF, Buxton JL. Obesity, genetic risk, and environment. BMJ. 2014;348.

51. Fava C, Sjogren M, Olsson S, Lovkvist H, Jood K, Engstrom G, et al. A genetic risk score for hypertension associates with the risk of ischemic stroke in a Swedish case-control study. Eur J Hum Genet. 2015;23(7):969-74.

52. Stegenga H, Haines A, Jones K, Wilding J. Identification, assessment, and management of overweight and obesity: summary of updated NICE guidance. BMJ : British Medical Journal. 2014;349:g6608.

53. Kaul S, Rothney MP, Peters DM, Wacker WK, Davis CE, Shapiro MD, et al. Dual-Energy X-Ray Absorptiometry for Quantification of Visceral Fat. Obesity (Silver Spring, Md). 2012;20(6):1313-8.

54. Micklesfield LK, Goedecke JH, Punyanitya M, Wilson KE, Kelly TL. Dual-energy X-ray performs as well as clinical computed tomography for the measurement of visceral fat. Obesity (Silver Spring). 2012;20(5):1109-14.

55. Ugale S, Gupta N, Modi KD, Kota SK, Satwalekar V, Naik V, et al. Prediction of remission after metabolic surgery using a novel scoring system in type 2 diabetes - a retrospective cohort study. J Diabetes Metab Disord. 2014;13(1):89-.

For peer review only

56. Aron-Wisnewsky J, Sokolovska N, Liu Y, Comaneshter DS, Vinker S, Pecht T, et al. The advanced-DiaRem score improves prediction of diabetes remission 1 year post-Roux-en-Y gastric bypass. Diabetologia. 2017;60(10):1892-902.

57. Hayes MT, Hunt LA, Foo J, Tychinskaya Y, Stubbs RS. A model for predicting the resolution of type 2 diabetes in severely obese subjects following Roux-en Y gastric bypass surgery. Obesity surgery. 2011;21(7):910-6.

58. Lee WJ, Chong K, Chen SC, Zachariah J, Ser KH, Lee YC, et al. Preoperative Prediction of Type 2 Diabetes Remission After Gastric Bypass Surgery: a Comparison of DiaRem Scores and ABCD Scores. Obesity surgery. 2016;26(10):2418-24.

59. Dixon JB, Hur KY, Lee WJ, Kim MJ, Chong K, Chen SC, et al. Gastric bypass in Type 2 diabetes with BMI < 30: weight and weight loss have a major influence on outcomes. Diabetic medicine : a journal of the British Diabetic Association. 2013;30(4):e127-34.

60. Dixon JB, Chuang LM, Chong K, Chen SC, Lambert GW, Straznicky NE, et al. Predicting the glycemic response to gastric bypass surgery in patients with type 2 diabetes. Diabetes care. 2013;36(1):20-6.

61. Robert M, Ferrand-Gaillard C, Disse E, Espalieu P, Simon C, Laville M, et al. Predictive factors of type 2 diabetes remission 1 year after bariatric surgery: impact of surgical techniques. Obesity surgery. 2013;23(6):770-5.

62. Pucci A, Tymoszuk U, Cheung WH, Makaronidis JM, Scholes S, Tharakan G, et al. Type 2 diabetes remission 2 years post Roux-en-Y gastric bypass and sleeve gastrectomy: the role of the weight loss and comparison of DiaRem and DiaBetter scores. Diabetic medicine : a journal of the British Diabetic Association. 2018;35(3):360-7.

63. Debedat J, Sokolovska N, Coupaye M, Panunzi S, Chakaroun R, Genser L, et al. Long-term Relapse of Type 2 Diabetes After Roux-en-Y Gastric Bypass: Prediction and Clinical Relevance. Diabetes care. 2018;41(10):2086-95.

For peer review only

64. Aminian A, Brethauer SA, Andalib A, Nowacki AS, Jimenez A, Corcelles R, et al. Individualized Metabolic Surgery Score: Procedure Selection Based on Diabetes Severity. Annals of surgery. 2017;266(4):650-7.

65. Bhasker AG, Remedios C, Batra P, Sood A, Shaikh S, Lakdawala M. Predictors of Remission of T2DM and Metabolic Effects after Laparoscopic Roux-en-y Gastric Bypass in Obese Indian Diabetics-a 5-Year Study. Obesity surgery. 2015;25(7):1191-7.

66. Hamza N, Abbas MH, Darwish A, Shafeek Z, New J, Ammori BJ. Predictors of remission of type 2 diabetes mellitus after laparoscopic gastric banding and bypass. Surgery for obesity and related diseases : official journal of the American Society for Bariatric Surgery. 2011;7(6):691-6.

67. Blackstone R, Bunt JC, Cortes MC, Sugerman HJ. Type 2 diabetes after gastric bypass: remission in five models using HbA1c, fasting blood glucose, and medication status. Surgery for obesity and related diseases : official journal of the American Society for Bariatric Surgery. 2012;8(5):548-55.

68. Schauer PR, Bhatt DL, Kirwan JP, Wolski K, Brethauer SA, Navaneethan SD, et al. Bariatric Surgery versus Intensive Medical Therapy for Diabetes — 3-Year Outcomes. New England Journal of Medicine. 2014;370(21):2002-13.

69. Hill NR, Levy JC, Matthews DR. Expansion of the homeostasis model assessment of β-cell function and insulin resistance to enable clinical trial outcome modeling through the interactive adjustment of physiology and treatment effects: iHOMA2. Diabetes care. 2013;36(8):2324-30.

70. Ali Aminian M, Sangeeta Kashyap M, David Cummings M, Deepa V Cherla M, Philip Schauer M, Roman Vangoitsenhoven MP. Presence Of Liver Steatosis Is Associated With Greater Diabetes Remission After Gastric Bypass Surgery. Obesity Week Conference 2019.

71. Steven S, Hollingsworth KG, Small PK, Woodcock SA, Pucci A, Aribisala B, et al. Weight Loss Decreases Excess Pancreatic Triacylglycerol Specifically in Type 2 Diabetes. Diabetes care. 2016;39(1):158-65.

For peer review only

AUTHOR CONTRIBUTIONS JSK, TT, SRB, AB, AA, SP and AP developed the protocol. JSK drafted the paper and TT, SRB, AB, AA, SP, KM and AP provided critical revisions.

FUNDING This work was supported by NIHR grant number [DRF-2017-10-042]. JSK is funded by a National Institute for Health Research (NIHR) Doctoral Research Fellowship for this research project. The views expressed are those of the author(s) and not necessarily those of the NIHR or the Department of Health and Social Care.

CONFLICTS OF INTERESTS STATEMENT. None declared

ETHICS APPROVAL This study was approved by the London Bloomsbury Ethics Committee (REC reference number 18/LO/0931).

For peer review only

BMJ Open

Who will benefit from bariatric surgery for diabetes? A protocol for an observational cohort study

Journal:	*BMJ Open*
Manuscript ID	bmjopen-2020-042355.R1
Article Type:	Protocol
Date Submitted by the Author:	07-Dec-2020
Complete List of Authors:	Kenkre, Julia; Imperial College London, Department of Metabolism, Digestion and Reproduction<br>Ahmed, Ahmed; Imperial College London, Department of Surgery and Cancer<br>purkayastha, sanjay; Imperial College London, Department of Surgery and Cancer<br>Malallah, Khalefah; Imperial College London, Department of Surgery and Cancer<br>Bloom, Stephen; Imperial College London, Clinical Director, North West London Pathology, Head of Division of Diabetes, Endocrinology and Metabolism, Imperial College, 6th Floor Commonwealth Building, Hammersmith Hospital, Imperial College London, Du Cane Road, London, W12 0NN<br>Blakemore, Alexandra; Brunel University College of Health and Life Sciences, Department of Life Sciences<br>Prevost, A; King's College London, Nightingale-Saunders Clinical Trials and Epidemiology Unit, King’s Clinical Trials Unit<br>Tan, Tricia; Imperial College London, Department of Metabolism, Digestion and Reproduction
**Primary Subject Heading**:	Diabetes and endocrinology
Secondary Subject Heading:	Surgery
Keywords:	General diabetes < DIABETES & ENDOCRINOLOGY, Adult surgery < SURGERY, NUTRITION & DIETETICS

SCHOLARONE™
Manuscripts

I, the Submitting Author has the right to grant and does grant on behalf of all authors of the Work (as defined in the below author licence), an exclusive licence and/or a non-exclusive licence for contributions from authors who are: i) UK Crown employees; ii) where BMJ has agreed a CC-BY licence shall apply, and/or iii) in accordance with the terms applicable for US Federal Government officers or employees acting as part of their official duties; on a worldwide, perpetual, irrevocable, royalty-free basis to BMJ Publishing Group Ltd ("BMJ") its licensees and where the relevant Journal is co-owned by BMJ to the co-owners of the Journal, to publish the Work in this journal and any other BMJ products and to exploit all rights, as set out in our licence.

The Submitting Author accepts and understands that any supply made under these terms is made by BMJ to the Submitting Author unless you are acting as an employee on behalf of your employer or a postgraduate student of an affiliated institution which is paying any applicable article publishing charge ("APC") for Open Access articles. Where the Submitting Author wishes to make the Work available on an Open Access basis (and intends to pay the relevant APC), the terms of reuse of such Open Access shall be governed by a Creative Commons licence – details of these licences and which Creative Commons licence will apply to this Work are set out in our licence referred to above.

Other than as permitted in any relevant BMJ Author's Self Archiving Policies, I confirm this Work has not been accepted for publication elsewhere, is not being considered for publication elsewhere and does not duplicate material already published. I confirm all authors consent to publication of this Work and authorise the granting of this licence.

For peer review only

TITLE PAGE

TITLE: Who will benefit from bariatric surgery for diabetes? A protocol for an observational cohort study.

Authors:

Dr Julia Kenkre, [1]

Mr Ahmed Ahmed[4]

Mr Sanjay Purkayastha[4]

Dr Khalefah Malallah [4]

Prof Stephen Bloom, [1]

Prof Alexandra Blakemore, [2]

Prof A. Toby Prevost, [3]

Prof Tricia Tan[1]

Author affiliations

1. Section of Endocrinology and Investigative Medicine, Imperial College, London. UK
2. Department of Life Sciences, Brunel University, London. UK
3. Nightingale-Saunders Clinical Trials and Epidemiology Unit, King's Clinical Trials Unit, King's College, London. UK
4. Department of Surgery and Cancer, Imperial College, London. UK

For peer review only - http://bmjopen.bmj.com/site/about/guidelines.xhtml

For peer review only

Corresponding author Tricia Tan, Section of Endocrinology and Investigative Medicine, 6th Floor Commonwealth Building, Imperial College Hammersmith Hospital Campus, Du Cane Road, London, W12 0NN. t.tan@imperial.ac.uk

WORD COUNT 2963

For peer review only

For peer review only - http://bmjopen.bmj.com/site/about/guidelines.xhtml

ABSTRACT

Introduction Type 2 diabetes mellitus (T2DM) and obesity are pandemic diseases that lead to a great deal of morbidity and mortality. The most effective treatment for obesity and T2DM is bariatric surgery; it can lead to long-term diabetes remission with 4 in 10 of those undergoing surgery having normal blood glucose on no medication one year post-operatively. However, surgery carries risks and, additionally, due to resource-limitation, there is a restricted number of patients who can access this treatment. Moreover, not all those who undertake surgery respond equally well metabolically. The objective of the current research is to prospectively investigate predictors of T2DM response following bariatric surgery, including those directly involved in its aetiopathogenesis such as fat distribution and genetic variants. This will inform development of a clinically applicable model to help prioritise this therapy to those predicted to have remission.

Methods and analysis A prospective multicentre observational cohort study of adult patients with T2DM and obesity undergoing Roux-en-Y gastric bypass (RYGB) surgery. Patients will be comprehensively assessed before surgery to determine their clinical, metabolic, psychological, genetic and fat distribution profiles. A multivariate logistic regression model will be used to assess the value of the factors derived from the pre-operative assessment in terms of prediction of diabetes remission.

Ethics and dissemination. Formal ethics review was undertaken with a favourable opinion (UK HRA RES reference number 18/LO/0931). The dissemination plan is to

For peer review only - http://bmjopen.bmj.com/site/about/guidelines.xhtml

present the results at conferences, in peer-reviewed journals as well as lay media and to patient organisations.

Trial registration number:

ClinicalTrials.gov Identifier: NCT03842475

ARTICLE SUMMARY

Strengths and limitations of this study

- One of the first studies to prospectively and comprehensively profile patients with type 2 diabetes undergoing bariatric surgery to assess predictors of diabetes remission.
- Collects prospective data on factors known to be involved in the aetiopathogenesis of T2DM such as genetics and fat distribution.
- A biorepository of tissue samples will be collected to allow multi-omics analysis.
- Long-term follow up for up to ten years will enable assessment of the durability of remission.
- The primary limitation is that this is a preliminary study; it will identify and internally validate predictors and will require further external validation in other cohorts.

KEY WORDS

Obesity, Bariatric Surgery, Diabetes Mellitus, Type 2

For peer review only - http://bmjopen.bmj.com/site/about/guidelines.xhtml

INTRODUCTION

Currently the majority of adults globally are obese or overweight. The increasing number of those with obesity is closely mirrored by the increasing prevalence of diabetes; currently over 400 million adults are estimated to be affected, 90% of whom have T2DM. (1) The close relationship between obesity, particularly 'central' or abdominal obesity, and T2DM has now been shown in multiple studies. (2, 3) Obesity, as defined by ethnicity-specific body mass index (BMI) cut-offs, is the principal modifiable risk factor for developing T2DM. However, the weight threshold at which T2DM develops varies between individuals, with significant variation amongst different ethnicities exemplifying two of the important factors that are thought to influence risk: individual pattern of where fat is deposited and underlying genetic predisposition.(4-9)

Epidemiological studies have shown those with a higher waist: hip ratio (WHR), representing centrally-deposited fat, are more likely to develop the disease.(3) Central fat or higher intra-abdominal visceral fat deposition is known to be metabolically more damaging with increased likelihood of developing abnormalities in glycaemia, blood pressure and lipid profiles.(10) However, it is not simply central adiposity but also ectopic fat that may have pathophysiological consequences.(11) Evidence is accumulating regarding the detrimental effect on glycaemia from fat ectopically deposited within liver, skeletal muscle and pancreas.(12)

T2DM shows heritability and many pathogenic variants have been discovered which influence β cell functioning and insulin resistance.(13, 14) Moreover, genetic susceptibility to T2DM is known to be correlated with phenotypic changes in fat distribution: genetic variants associated with insulin resistance have been found to be correlated with lower subcutaneous adipose tissue (SAT) and a genetic predisposition

For peer review only - http://bmjopen.bmj.com/site/about/guidelines.xhtml

to diabetes has been found in those with impaired SAT adipogenesis and increased waist circumference.(15) Combining GWAS data to derive personalised genetic risk, whilst challenging, has been successfully achieved in other conditions with the potential to add further precision to a predictive score. (16, 17)

Remission of diabetes

The most effective treatment for obesity and diabetes is bariatric surgery; it has been relabelled as 'metabolic surgery' to highlight its primary utility as a therapy for diabetes and other related metabolic dysfunctions, including dyslipidaemia and non-alcoholic fatty liver disease, as opposed to primarily treating excess weight.(18) Randomised control trials have shown bariatric surgery consistently out performs best-medical therapy in improving glycaemic control and leads to durable remission in a significant proportion.(19, 20) RYGB, the gold-standard procedure for those with diabetes, involves anastomosis of a small pouch of stomach to jejunum such that food is diverted to the jejunum, bypassing the majority of the stomach and the duodenum. Following RYGB, remission of diabetes (as defined by markers of normoglycaemia in the absence of anti-diabetic medication) occurs in 4 in 10 patients at one year and this remission is long-lasting: the majority of these patients remain in remission at 5 years.(18-20) Furthermore, surgery also leads to a reduction in micro- and macrovascular complications associated with diabetes.(21)

No single unifying mechanism found thus far can explain the improvement in glycaemic control seen in those with T2DM following RYGB. Whilst it is clear that the 25-30% weight loss seen by 12-18 months post-operatively plays a significant role, improvement in glycaemia occurs within days following surgery, prior to any significant weight reduction, suggesting a role for weight-loss independent mechanisms.(22)

For peer review only - http://bmjopen.bmj.com/site/about/guidelines.xhtml

Reduced calorie intake post-surgery is one such putative weight-loss independent mechanism for the immediate improvement in glycaemia as those on a very low-calorie diet can achieve reversal of the metabolic features of diabetes.(23) However, caloric restriction normally also leads to homeostatic compensation or counter-regulation including increased appetite, reduced energy expenditure and secretion of orexigenic gut hormones. In contrast, following surgery, appetite is known to reduce and weight-adjusted resting energy expenditure may increase. (24-26)

It is likely that caloric restriction and weight loss are only two of many mechanisms that contribute to the post-operative metabolic improvement seen. Insulinotropic and anorexigenic gut hormones including glucagon like peptide 1 (GLP-1), Peptide YY (PYY) and oxyntomodulin increase following surgery.(27, 28) PYY and GLP-1 have been implicated in the restoration of insulin secretion after RYGB.(29, 30) Furthermore, infusion of such hormones leads to improved metabolic control.(31) Changes in their secretion have been correlated with post-operative metabolic and weight loss response. (30, 32, 33) Associated with these findings are the increased levels of plasma bile acids after surgery which have been correlated with increased levels of GLP-1.(34) Bile acids have been implicated in the improvement in glycaemia via signalling through the nuclear farnesoid X receptor (FXR) and G-protein-coupled TGR5 receptors.(35, 36) TGR5 activation leads to increased secretion of insulinotropic GLP-1 and modulates insulin sensitivity in tissues such as muscle and brown adipose tissue.(37) Whilst changes in bile acids play a role in metabolic improvement, this still remains an area of active research.

Further adding to the complexity of the mechanism of remission, differences in the gut microbiota i.e. the microorganisms commensal within the gut, have been seen between

For peer review only - http://bmjopen.bmj.com/site/about/guidelines.xhtml

lean and obese individuals.(38) There is some evidence that, as a regulator of the host's metabolism, the gut microbiota may be associated with obesity through several mechanisms such as enhancement of fat storage.(39) Significant changes occur in the gut microbiota following surgery (40) but the causal relationship between the microbiotal changes and metabolic improvements, i.e. whether the metabolic improvements come from the microbiotal changes or vice versa, has yet to be established. Furthermore, there is a bi-directional relationship between the microbiome and bile acids: whilst the gut microbiota have a role in converting primary to secondary bile acids, bile acids can affect the diversity of gut microbiota.(41)

We conclude that to explore the mechanism of remission further a comprehensive multi-omics approach must therefore be employed.

Predicting diabetes remission following surgery

There is a substantial disparity between the numbers eligible for bariatric surgery and the resources available to deliver it; in the UK two million people fulfil the criteria but surgery is only being performed in c. 5600 patients per year.(42) Furthermore, there is variation in the metabolic response between individuals such that less than half are expected to have complete diabetes remission at one year following surgery. If we can better predict who will benefit from bariatric surgery by remission of diabetes we can better inform patients and direct the surgery to where it will be most effective.(43) Currently in the UK, BMI cut-offs dictate eligibility for surgery, along with more recently introduced diabetes duration and ethnicity criteria.(44) However, baseline BMI does not correlate well with the likelihood of diabetes remission thus other predictors are necessary to better inform eligibility based on intended benefit.(45)

For peer review only - http://bmjopen.bmj.com/site/about/guidelines.xhtml

Predictive factors have been found in multiple, mainly retrospective studies and some have been incorporated into models of diabetes remission.(46-48) These models have tried to predict diabetes remission based on easily available clinical data making them translatable to everyday practice. However, they remain limited in their clinical applicability.(49) Moreover, none have incorporated fat distribution or genetic risk factors which have important aetiopathological roles in T2DM. Furthermore, they are, in most cases, based on retrospective studies. One problem faced in this instance is that the consensus definition of diabetes remission depends on withdrawal of pharmacological therapies for T2DM for one year. There is often no standardised pathway to assess the remission status after surgery, which can lead to differing practice amongst clinicians thus hampering the ability to define remission status with accuracy.

This study will use genetic profiling, identifying genetic variants associated with fat distribution patterns, β-cell dysfunction, T2DM or insulin resistance, combined into an individual genetic-risk score for each patient. (50, 51) Moreover, it will assess the distribution pattern of total body fat in addition to ectopic fat deposition within the liver, muscle and pancreas. These variables will be tested to assess if they are predictive of diabetes remission. In addition, patients will be profiled for variation in biomarkers postulated to be mechanistically involved in diabetes remission including gut hormones, microbiotal changes and bile acids.

The ultimate aim of the study will be identify novel covariates predictive of diabetes remission after bariatric surgery, with the intention that these can be further investigated for their predictive power in larger studies and other populations.

OBJECTIVES

For peer review only - http://bmjopen.bmj.com/site/about/guidelines.xhtml

Primary objective

To develop and internally validate a predictive model of diabetes remission following bariatric surgery.

Secondary objectives

To assess changes in diabetes and obesity-related co-morbidities following surgery and investigate mechanism of diabetes remission.

METHODS AND ANALYSIS

This is a prospective, multicentre observational cohort study investigating predictors of diabetes remission in patients with diabetes/prediabetes undergoing Roux-en-y gastric bypass surgery with standardised biliopancreatic and alimentary limb lengths of 50 cm and 100 cm respectively.

Participants and selection criteria

Study participants will be recruited according the inclusion and exclusion criteria detailed in Table 1.

**Inclusion criteria**	Patients with prediabetes or diabetes eligible for bariatric surgery on the NHS, i.e. BMI ≥35 with a co-morbidity or ≥40, lower BMIs accepted if of Asian origin or recent-onset diabetes, referred to Imperial Weight Centre or Chelsea and Westminster NHS Trust(44, 52)
	Stable weight (≤ 10% variation) for at least 3 months
**Exclusion criteria**	Current pregnancy
	Inability to give informed consent

For peer review only - http://bmjopen.bmj.com/site/about/guidelines.xhtml

	Type 1 diabetes, low fasting C-peptide, secondary diabetes or absence of β-cell function
	Unable to undergo DEXA
	Cirrhosis, ascites, or other condition that may modify body fat composition e.g. underlying malignancy
	Participation in a concomitant interventional trial within the last 3 months that would affect the current study results or mean that undergoing the study would be too burdensome for the participant.
	Unable to understand English.

Table 1. Inclusion and exclusion criteria

For peer review only

Outcomes

The primary outcome is diabetes remission (partial and complete) or diabetes remission (complete) measured post-operatively at 15 months. This will be defined by American Diabetes Association criteria, such that complete remission is fasting glucose <5.6 mmol/L, HbA1c <42 mmol/mol on no anti-diabetic agents, and partial remission is defined as fasting glucose 5.6-6.9 mmol/L, HbA1c <48 mmol/mol on no anti-diabetic agents. Patients will be seen at 3 months as part of routine clinical follow-up and will have anti-diabetic medication stopped according to a standardised pathway. An assessment of diabetes remission will also be made at 1, 2, 3 and 5 years.

In addition to the primary outcome we will collect phenotyping data to allow assessment of diabetes severity and obesity associated co-morbidities.

Exploratory outcomes:

For peer review only - http://bmjopen.bmj.com/site/about/guidelines.xhtml

• Change in epigenetic data including DNA methylation, histone modifications and non-coding RNA in blood, liver, muscle, fat (subcutaneous and visceral) and stomach and small bowel and spermatozoa

• Changes in the transcriptome in blood, liver, muscle, fat (subcutaneous and visceral) and stomach and small bowel and spermatozoa

• Changes in gut hormone profiles in response to a mixed meal

• Histological scoring of specimens for NAFLD/NASH

• Changes in gut microbiotal diversity, relative abundance of selected phyla (e.g. Bacteroides and Firmicutes), and frequency of operational taxonomic units.

Sample size calculation

Using a logistic regression model to predict a 75% partial and completed diabetes remission rate from multiple predictors, a sample size of 150 provides 90% power at the two-sided 5% significance level to detect an odds ratio of 2.0 per standard deviation of any continuous predictor.(53) This allows for up to 10% of the variation in any one predictor to be explained by the others in the model, and 15% loss to follow-up. The area under ROC curve, overall performance measure, will be estimated with a 95% confidence interval width of at most +/-0.1 for an area above 0.65. With data analysed from 100 patients there would still be in excess of 80% power to detect the odds ratio of 2.0, and a precision of at most +/-0.12 for the ROC curve area. This is reasonable enough to allow some deviation in assumptions, such as the dropout rate.

Measures

For peer review only - http://bmjopen.bmj.com/site/about/guidelines.xhtml

For peer review only

Measures are summarised in Table 2.

Visit number	1	Surgery	2	3	4	5
**Month**	**Pre-op**<br>*Co-variates measured here*	**0**	**15**<br>*Primary outcome measured here*	**24**	**36**	**60**
**Clinical assessment. Anthropometrics* and bioimpedance measurement**	✓	Weight only	✓	✓	✓	✓
**Blood tests including Hb1c and fasting glucose to enable assessment of remission +**	✓	✓	✓	✓	✓	✓
**Mixed meal test (MMT)**	✓		✓			
**Psychological and exercise screening questionnaires**	✓		✓	✓	✓	✓
**Food intake assessed by a 4-7-day food diary**	✓		✓			✓
**24-hour blood pressure and capillary or continuous glucose monitoring**	✓		✓			
**Urine ** and stool sample**	✓	✓	✓			✓
**Fat biopsy**		✓	✓			

For peer review only - http://bmjopen.bmj.com/site/about/guidelines.xhtml

**Liver, muscle and gut biopsy**		✓				
**Whole body DEXA**	✓		✓			
**Sperm sample (Male participants only)**	✓		✓			
**Whole body MRI**	✓		✓			
**Endoscopy and colonoscopy + biopsy (Selected participants only)**	✓		✓			

Table 2. Time and Events table of measures that participants will undergo throughout the study. *Anthropometrics includes height, waist, hip and neck circumference. + Blood tests include glucose, insulin, C-peptide, gut hormones, free fatty acids, bile acids, plasma metabolomics, DNA, RNA, cortisol, HbA1c, lipids and FGF 19/21. ** Urine testing will include microalbumin:creatinine and metobolomic assessment.

In addition to collection of a full clinical assessment the following measures will be undertaken:

Mixed Meal test

Pre-operatively and at 15 months following their first study visit participants will undergo a mixed meal test, using a stimulus of 14 g protein, 12.9 g fat, 39.6 g carbohydrate, 330 kcal, 137.5 ml (Ensure Compact, Abbott). Prior to this, withdrawal of diabetes medications which will occur using a standardised pathway. Participants will

For peer review only - http://bmjopen.bmj.com/site/about/guidelines.xhtml

have blood samples taken to measure HbA1c, fasting and stimulated insulin/ C-peptide/ glucose, gut hormones, free fatty acids, bile acids, plasma metabolites and FGF-19/-21.

Questionnaires

The following questionnaires will be used to examine psychological wellbeing: Alcohol Use Disorders Identification Test (AUDIT), Eating Disorder Examination Questionnaire (EDEQ), Difficulty in Emotion Regulation Scale (DERS), Hospital Anxiety and Depression Scale (HADS) and Short Form 36 (SF-36). The Temperament and Character Inventory (TCI) will also be collected at baseline alone. The International Physical Activity Questionnaire (IPAQ) short form will be used to assess exercise levels.

Questionnaires will be analysed to assess if they impact on likelihood of obtaining remission and, in subsequent visits if they modify the consequences of surgery i.e. maintenance of diabetes remission. The TCI will be used to assess whether personality subtype predicts metabolic response to surgery.

Metabolic status

Lipid profile along with 24-hour blood pressure monitoring will be analysed to further assess the response of these cardiometabolic risk factors to surgery. Continuous glucose measurement sensors or capillary glucose readings will be undertaken to give an alternative measure of glycaemic control.

Body composition

All patients will undergo a whole body DEXA prior to and 15 months following surgery. This is a validated tool to estimate visceral adipose tissue.(54, 55)

For peer review only - http://bmjopen.bmj.com/site/about/guidelines.xhtml

Where feasible, participants will undergo a gold standard assessment of visceral adipose tissue using MRI which will also quantify liver, muscle and pancreatic fat.

DNA

Genomic DNA will be extracted from whole blood samples collected in an EDTA tube. A microarray customised to variants known to be associated with obesity, T2DM and fat patterning will be used to analyse samples. A weighted genetic risk score, with each variant weighted according to their previously published effect size,will be calculated individually .

Omics

These will be used for to explore possible mechanisms explaining the metabolic changes before and after surgery.

Transcriptomics/epigenomics/proteomics: Longitudinal tissue samples will be collected where possible and will undergo multi-omics assessment.

Microbiotal analysis: Longitudinal samples of stool will be collected to assess the microbiome.

Metabolomics: Longitudinal fasting urine and plasma metabolomic samples will be collected pre and following surgery

Predictors

Candidate predictors (for examples, see Table 3) have been selected based on those identified in previous retrospective cohort studies. Two unique predictors, fat

For peer review only - http://bmjopen.bmj.com/site/about/guidelines.xhtml

distribution and genetic risk score, will also be tested. We will then cautiously extend the model to address other factors in a univariate manner.

Predictor	Units/categories	Variable type	Source data	Previous studies
**Age**	years	Continuous	Clinical history	(46, 47, 56, 57)
**BMI or Weight**	kg/m^2 or kg	Continuous	V1	(58, 59)
**C peptide**	pmol/L	Continuous	Mixed meal test	(46, 56, 60)
**Diabetes duration**	years	Continuous	Clinical history	(46, 56, 57, 61-65)
**Fasting plasma glucose**	mmol/L	Continuous	Blood test	(66)
**Fat distribution**	Visceral fat and Android: Gynoid fat	Continuous	(DEXA, impedance, MRI)	
**Genetic risk score**	Weighted genetic risk score	Categorical	DNA V1	
**HbA1c with medications**	mmol/mol<br><br>Insulin, sulphonylureas, insulin sensitising	Continuous: will interact with medications since directly	Blood test	(47, 57, 58, 62-65, 67-69)

For peer review only - http://bmjopen.bmj.com/site/about/guidelines.xhtml

	agents, GLP-1 analogues, DPP-IV inhibitors, SGLT-2 inhibitors	affected by them		
**HOMA 2**	Using interactive 24-variable assessment of homeostatic model		Mixed meal test	(70)
**Hypertension**	Present/Absent<br><br>Controlled by 1-5 agents	Categorical	Medical history, 24-hour blood pressure monitoring	
**Insulinogenic index**	δ insulin (0-30 min) / δ glucose (0-30 min)	Continuous	Mixed meal test	
**Presence of fatty liver disease as assessed by non-invasive markers**	NAFLD liver fat score, Fatty Liver Index, FIB-4	Categorical	Medical history, MRI, Non-invasive markers including liver function tests	(71)
**Presence of diabetes**	Microvascular disease (including	Categorical	Clinical history,	(56)

For peer review only - http://bmjopen.bmj.com/site/about/guidelines.xhtml

**complications**	subgroups)<br><br>Macrovascular disease<br><br>(including subgroups)		microalbumin: creatinine, retinal imaging and clinical examination.	
**Presence of ectopic fat**	% liver fat<br><br>% muscle fat<br><br>% pancreatic fat	Continuous	MRI	(72)
**Sex**	Male/Female	Categorical	Clinical history	
**TCI-R scoring**	Novelty seeking, Harm avoidance, Reward Dependence, Persistence, Self-Directedness, Cooperativeness and Self-Transcendence	Continuous	V1	
**Waist: hip ratio**	Measured in cm	Continuous	V1	

Table 3. Candidate predictors of diabetes remission. V1 is study visit 1.

For peer review only - http://bmjopen.bmj.com/site/about/guidelines.xhtml

A logistic regression model predicting diabetes remission assessed at the 15-months visit will be built using the prospective cohort data. This was chosen in preference to an ordinal regression approach, such as the proportional odds model, because of the need to have a clear and independent interpretation of the predictive contribution both to complete remission and to complete and partial remission. Logistic regression was preferred over another alternative approach, discriminant analysis, for the same reason and because logistic regression provides predicted probabilities for individual patients which can then be then used within ROC curve analysis to provide estimates of sensitivity and specificity of the regression models measuring their accuracy to predict the diabetes remission outcome of bariatric surgery.

Initially, the effect of each of the clinical predictors and other covariates (patient demographic factors) of interest on each outcome is to be fitted in univariable models. Any clinical predictor that is significant at a threshold of p-value ≤0.20 will then be included in a multivariable model predicting the outcome of diabetes remission. Clinical predictors and other covariates will then be eliminated in stepwise fashion until each remaining predictor is significant at the p-value=0.05 threshold and the models are found to be stable. Pattern of fat storage will be measured by VAT mass, SAT mass and VAT/SAT ratio, and genes influencing diabetes risk and fat deposition will be measured by the Genetic Risk Score using the approach of Fava et al. (51) These will be added to the model, if not already included, to allow their association with diabetes remission to be formally quantified and tested. We will cautiously extend the model to address other factors in a univariate manor. Internal validation of the selection of the predictive model will be done using bootstrap sampling with re-application of the model selection procedure using at least 1000 replications. The performance of the prediction model

For peer review only - http://bmjopen.bmj.com/site/about/guidelines.xhtml

under different scenarios will be assessed, primarily by obtaining fitted probabilities of remission from the model to estimate the area under the ROC curve, and also by demonstrating the sensitivity, specificity, positive predictive value and negative predictive value across a series of cut points representing a range of scenarios including high sensitivity, high specificity and equivalent sensitivity and specificity.

Survival curves will be plotted to show the proportion remaining free from diabetes over the longer term according to categories of predictors identified to be important.

Patient and public involvement

Patients were consulted about the study objectives and selected study procedures to assess acceptability.

ETHICS AND DISSEMINATION:

This study complies with the Declaration of Helsinki and the principles of Good Clinical Practice. The researchers plan to disseminate results at conferences, in peer-reviewed journals as well as lay media and to patient organisations.

DATA STATEMENT

Anonymized data from the study will be made available upon request from the Principal Investigator upon reasonable request. ClinicalTrials.gov Identifier: NCT03842475

REFERENCES

1. Federation ID. IDF DIABETES ATLAS, Eight edition. 2017.
2. Neeland IJ, Turer AT, Ayers CR, et al. Dysfunctional adiposity and the risk of prediabetes and type 2 diabetes in obese adults. JAMA : the journal of the American Medical Association. 2012;308(11):1150-9.

For peer review only - http://bmjopen.bmj.com/site/about/guidelines.xhtml

3. The InterAct C. Long-Term Risk of Incident Type 2 Diabetes and Measures of Overall and Regional Obesity: The EPIC-InterAct Case-Cohort Study. PLoS Med. 2012;9(6):e1001230.
4. Taylor R. Pathogenesis of type 2 diabetes: tracing the reverse route from cure to cause. Diabetologia. 2008;51(10):1781-9.
5. Heymsfield SB, Peterson CM, Thomas DM, Heo M, Schuna JM, Jr. Why are there race/ethnic differences in adult body mass index-adiposity relationships? A quantitative critical review. Obesity reviews : an official journal of the International Association for the Study of Obesity. 2016;17(3):262-75.
6. Taylor R, Holman RR. Normal weight individuals who develop type 2 diabetes: the personal fat threshold. Clin Sci (Lond). 2015;128(7):405-10.
7. Wildman RP, Muntner P, Reynolds K, McGinn AP, Rajpathak S, Wylie-Rosett J, et al. The Obese Without Cardiometabolic Risk Factor Clustering and the Normal Weight With Cardiometabolic Risk Factor Clustering: Prevalence and Correlates of 2 Phenotypes Among the US Population (NHANES 1999-2004). JAMA internal medicine. 2008;168(15):1617-24.
8. Bouchard C, Tremblay A, Despres JP, Nadeau A, Lupien PJ, Theriault G, et al. The response to long-term overfeeding in identical twins. The New England journal of medicine. 1990;322(21):1477-82.
9. Shai I, Jiang R, Manson JE, Stampfer MJ, Willett WC, Colditz GA, et al. Ethnicity, Obesity, and Risk of Type 2 Diabetes in Women. A 20-year follow-up study. 2006;29(7):1585-90.
10. Despres JP, Lemieux I. Abdominal obesity and metabolic syndrome. Nature. 2006;444(7121):881-7.
11. Ferrara D, Montecucco F, Dallegri F, Carbone F. Impact of different ectopic fat depots on cardiovascular and metabolic diseases. Journal of cellular physiology. 2019.
12. Jaghutriz B, Wagner R, Machann J, Stefan N, Peter A, Siegel-Axel DI, et al. Association of Pancreas Fat with Impaired Insulin Secretion Depends on Liver Fat and Circulating Fatty Acids. Diabetes. 2018;67(Supplement 1):1819-P.
13. Langenberg C, Lotta LA. Genomic insights into the causes of type 2 diabetes. The Lancet. 2018;391(10138):2463-74.
14. Fuchsberger C, Flannick J, Teslovich TM, Mahajan A, Agarwala V, Gaulton KJ, et al. The genetic architecture of type 2 diabetes. Nature. 2016;536(7614):41-7.
15. Shungin D, Winkler TW, Croteau-Chonka DC, Ferreira T, Locke AE, Mägi R, et al. New genetic loci link adipose and insulin biology to body fat distribution. Nature. 2015;518(7538):187-96.
16. Pedersen HK, Gudmundsdottir V, Pedersen MK, Brorsson C, Brunak S, Gupta R. Ranking factors involved in diabetes remission after bariatric surgery using machine-learning integrating clinical and genomic biomarkers. Npj Genomic Medicine. 2016;1:16035.
17. Khera AV, Chaffin M, Aragam KG, Haas ME, Roselli C, Choi SH, et al. Genome-wide polygenic scores for common diseases identify individuals with risk equivalent to monogenic mutations. Nature genetics. 2018;50(9):1219-24.
18. Schauer PR, Bhatt DL, Kirwan JP, Wolski K, Aminian A, Brethauer SA, et al. Bariatric Surgery versus Intensive Medical Therapy for Diabetes — 5-Year Outcomes. New England Journal of Medicine. 2017;376(7):641-51.
19. Schauer PR, Kashyap SR, Wolski K, Brethauer SA, Kirwan JP, Pothier CE, et al. Bariatric Surgery versus Intensive Medical Therapy in Obese Patients with Diabetes. New England Journal of Medicine. 2012;366(17):1567-76.
20. Mingrone G, Panunzi S, De Gaetano A, Guidone C, Iaconelli A, Leccesi L, et al. Bariatric surgery versus conventional medical therapy for type 2 diabetes. The New England journal of medicine. 2012;366(17):1577-85.
21. Sjostrom L, Peltonen M, Jacobson P, Ahlin S, Andersson-Assarsson J, Anveden A, et al. Association of bariatric surgery with long-term remission of type 2 diabetes and with microvascular and macrovascular complications. JAMA : the journal of the American Medical Association. 2014;311(22):2297-304.

For peer review only - http://bmjopen.bmj.com/site/about/guidelines.xhtml

For peer review only

22. Courcoulas AP, Christian NJ, Belle SH, Berk PD, Flum DR, Garcia L, et al. Weight change and health outcomes at 3 years after bariatric surgery among individuals with severe obesity. JAMA : the journal of the American Medical Association. 2013;310(22):2416-25.
23. Lim EL, Hollingsworth KG, Aribisala BS, Chen MJ, Mathers JC, Taylor R. Reversal of type 2 diabetes: normalisation of beta cell function in association with decreased pancreas and liver triacylglycerol. Diabetologia. 2011;54(10):2506-14.
24. Al-Najim W, Docherty NG, le Roux CW. Food Intake and Eating Behavior After Bariatric Surgery. Physiol Rev. 2018;98(3):1113-41.
25. Faria SL, Faria OP, Buffington C, de Almeida Cardeal M, Rodrigues de Gouvea H. Energy expenditure before and after Roux-en-Y gastric bypass. Obesity surgery. 2012;22(9):1450-5.
26. Wilms B, Ernst B, Thurnheer M, Schmid SM, Spengler CM, Schultes B. Resting energy expenditure after Roux-en Y gastric bypass surgery. Surgery for obesity and related diseases : official journal of the American Society for Bariatric Surgery. 2018;14(2):191-9.
27. Laferrère B. Diabetes remission after bariatric surgery: is it just the incretins? International journal of obesity (2005). 2011;35 Suppl 3(0 3):S22-S5.
28. Dirksen C, Jorgensen NB, Bojsen-Moller KN, Jacobsen SH, Hansen DL, Worm D, et al. Mechanisms of improved glycaemic control after Roux-en-Y gastric bypass. Diabetologia. 2012;55(7):1890-901.
29. Ramracheya Reshma D, McCulloch Laura J, Clark A, Wiggins D, Johannessen H, Olsen Magnus K, et al. PYY-Dependent Restoration of Impaired Insulin and Glucagon Secretion in Type 2 Diabetes following Roux-En-Y Gastric Bypass Surgery. Cell Reports.15(5):944-50.
30. Nannipieri M, Baldi S, Mari A, Colligiani D, Guarino D, Camastra S, et al. Roux-en-Y Gastric Bypass and Sleeve Gastrectomy: Mechanisms of Diabetes Remission and Role of Gut Hormones. The Journal of Clinical Endocrinology & Metabolism. 2013;98(11):4391-9.
31. Torekov SS, Kipnes MS, Harley RE, Holst JJ, Ehlers MR. Dose response of subcutaneous GLP-1 infusion in patients with type 2 diabetes. Diabetes, obesity & metabolism. 2011;13(7):639-43.
32. le Roux CW, Welbourn R, Werling M, Osborne A, Kokkinos A, Laurenius A, et al. Gut hormones as mediators of appetite and weight loss after Roux-en-Y gastric bypass. Annals of surgery. 2007;246(5):780-5.
33. Nielsen MS, Ritz C, Wewer Albrechtsen NJ, Holst JJ, le Roux CW, Sjödin A. Oxyntomodulin and glicentin may predict the effect of bariatric surgery on food preferences and weight loss. The Journal of Clinical Endocrinology & Metabolism. 2020.
34. Kaska L, Sledzinski T, Chomiczewska A, Dettlaff-Pokora A, Swierczynski J. Improved glucose metabolism following bariatric surgery is associated with increased circulating bile acid concentrations and remodeling of the gut microbiome. World journal of gastroenterology. 2016;22(39):8698-719.
35. Patti ME, Houten SM, Bianco AC, Bernier R, Larsen PR, Holst JJ, et al. Serum bile acids are higher in humans with prior gastric bypass: potential contribution to improved glucose and lipid metabolism. Obesity (Silver Spring). 2009;17(9):1671-7.
36. Gerhard GS, Styer AM, Wood GC, Roesch SL, Petrick AT, Gabrielsen J, et al. A role for fibroblast growth factor 19 and bile acids in diabetes remission after Roux-en-Y gastric bypass. Diabetes care. 2013;36(7):1859-64.
37. Wang W, Cheng Z, Wang Y, Dai Y, Zhang X, Hu S. Role of Bile Acids in Bariatric Surgery. Front Physiol. 2019;10:374.
38. Turnbaugh PJ, Hamady M, Yatsunenko T, Cantarel BL, Duncan A, Ley RE, et al. A core gut microbiome in obese and lean twins. Nature. 2009;457(7228):480-4.
39. Magouliotis DE, Tasiopoulou VS, Sioka E, Chatedaki C, Zacharoulis D. Impact of Bariatric Surgery on Metabolic and Gut Microbiota Profile: a Systematic Review and Meta-analysis. Obesity surgery. 2017.
40. Li JV, Ashrafian H, Bueter M, Kinross J, Sands C, le Roux CW, et al. Metabolic surgery profoundly influences gut microbial-host metabolic cross-talk. Gut. 2011;60(9):1214-23.

For peer review only - http://bmjopen.bmj.com/site/about/guidelines.xhtml

41. Cani PD, Van Hul M, Lefort C, Depommier C, Rastelli M, Everard A. Microbial regulation of organismal energy homeostasis. Nature Metabolism. 2019;1(1):34-46.
42. Society BOaMS. Clinical Outcomes 2017-18. 2018.
43. Ahmad A, Laverty AA, Aasheim E, Majeed A, Millett C, Saxena S. Eligibility for bariatric surgery among adults in England: analysis of a national cross-sectional survey. JRSM Open. 2014;5(1).
44. NICE. Obesity: identification, assessment and management of overweight and obesity in children, young people and adult. 2014.
45. Panunzi S, De Gaetano A, Carnicelli A, Mingrone G. Predictors of remission of diabetes mellitus in severely obese individuals undergoing bariatric surgery: do BMI or procedure choice matter? A meta-analysis. Annals of surgery. 2015;261(3):459-67.
46. Lee WJ, Hur KY, Lakadawala M, Kasama K, Wong SK, Chen SC, et al. Predicting success of metabolic surgery: age, body mass index, C-peptide, and duration score. Surgery for obesity and related diseases : official journal of the American Society for Bariatric Surgery. 2013;9(3):379-84.
47. Still CD, Wood GC, Benotti P, Petrick AT, Gabrielsen J, Strodel WE, et al. Preoperative prediction of type 2 diabetes remission after Roux-en-Y gastric bypass surgery: a retrospective cohort study. The lancet Diabetes & endocrinology. 2014;2(1):38-45.
48. Zhang R, Borisenko O, Telegina I, Hargreaves J, Ahmed AR, Sanchez Santos R, et al. Systematic review of risk prediction models for diabetes after bariatric surgery. BJS. 2016;103(11):1420-7.
49. Tharakan G, Scott R, Szepietowski O, Miras AD, Blakemore AI, Purkayastha S, et al. Limitations of the DiaRem Score in Predicting Remission of Diabetes Following Roux-En-Y Gastric Bypass (RYGB) in an ethnically Diverse Population from a Single Institution in the UK. Obesity surgery. 2016.
50. Blakemore AIF, Buxton JL. Obesity, genetic risk, and environment. BMJ. 2014;348.
51. Fava C, Sjogren M, Olsson S, Lovkvist H, Jood K, Engstrom G, et al. A genetic risk score for hypertension associates with the risk of ischemic stroke in a Swedish case-control study. Eur J Hum Genet. 2015;23(7):969-74.
52. Stegenga H, Haines A, Jones K, Wilding J. Identification, assessment, and management of overweight and obesity: summary of updated NICE guidance. BMJ : British Medical Journal. 2014;349:g6608.
53. Hsieh FY, Bloch DA, Larsen MD. A simple method of sample size calculation for linear and logistic regression. Statistics in medicine. 1998;17(14):1623-34.
54. Kaul S, Rothney MP, Peters DM, Wacker WK, Davis CE, Shapiro MD, et al. Dual-Energy X-Ray Absorptiometry for Quantification of Visceral Fat. Obesity (Silver Spring, Md). 2012;20(6):1313-8.
55. Micklesfield LK, Goedecke JH, Punyanitya M, Wilson KE, Kelly TL. Dual-energy X-ray performs as well as clinical computed tomography for the measurement of visceral fat. Obesity (Silver Spring). 2012;20(5):1109-14.
56. Ugale S, Gupta N, Modi KD, Kota SK, Satwalekar V, Naik V, et al. Prediction of remission after metabolic surgery using a novel scoring system in type 2 diabetes - a retrospective cohort study. J Diabetes Metab Disord. 2014;13(1):89-.
57. Aron-Wisnewsky J, Sokolovska N, Liu Y, Comaneshter DS, Vinker S, Pecht T, et al. The advanced-DiaRem score improves prediction of diabetes remission 1 year post-Roux-en-Y gastric bypass. Diabetologia. 2017;60(10):1892-902.
58. Hayes MT, Hunt LA, Foo J, Tychinskaya Y, Stubbs RS. A model for predicting the resolution of type 2 diabetes in severely obese subjects following Roux-en Y gastric bypass surgery. Obesity surgery. 2011;21(7):910-6.
59. Lee WJ, Chong K, Chen SC, Zachariah J, Ser KH, Lee YC, et al. Preoperative Prediction of Type 2 Diabetes Remission After Gastric Bypass Surgery: a Comparison of DiaRem Scores and ABCD Scores. Obesity surgery. 2016;26(10):2418-24.

For peer review only - http://bmjopen.bmj.com/site/about/guidelines.xhtml

60. Dixon JB, Hur KY, Lee WJ, Kim MJ, Chong K, Chen SC, et al. Gastric bypass in Type 2 diabetes with BMI < 30: weight and weight loss have a major influence on outcomes. Diabetic medicine : a journal of the British Diabetic Association. 2013;30(4):e127-34.
61. Dixon JB, Chuang LM, Chong K, Chen SC, Lambert GW, Straznicky NE, et al. Predicting the glycemic response to gastric bypass surgery in patients with type 2 diabetes. Diabetes care. 2013;36(1):20-6.
62. Robert M, Ferrand-Gaillard C, Disse E, Espalieu P, Simon C, Laville M, et al. Predictive factors of type 2 diabetes remission 1 year after bariatric surgery: impact of surgical techniques. Obesity surgery. 2013;23(6):770-5.
63. Pucci A, Tymoszuk U, Cheung WH, Makaronidis JM, Scholes S, Tharakan G, et al. Type 2 diabetes remission 2 years post Roux-en-Y gastric bypass and sleeve gastrectomy: the role of the weight loss and comparison of DiaRem and DiaBetter scores. Diabetic medicine : a journal of the British Diabetic Association. 2018;35(3):360-7.
64. Debedat J, Sokolovska N, Coupaye M, Panunzi S, Chakaroun R, Genser L, et al. Long-term Relapse of Type 2 Diabetes After Roux-en-Y Gastric Bypass: Prediction and Clinical Relevance. Diabetes care. 2018;41(10):2086-95.
65. Aminian A, Brethauer SA, Andalib A, Nowacki AS, Jimenez A, Corcelles R, et al. Individualized Metabolic Surgery Score: Procedure Selection Based on Diabetes Severity. Annals of surgery. 2017;266(4):650-7.
66. Bhasker AG, Remedios C, Batra P, Sood A, Shaikh S, Lakdawala M. Predictors of Remission of T2DM and Metabolic Effects after Laparoscopic Roux-en-y Gastric Bypass in Obese Indian Diabetics-a 5-Year Study. Obesity surgery. 2015;25(7):1191-7.
67. Hamza N, Abbas MH, Darwish A, Shafeek Z, New J, Ammori BJ. Predictors of remission of type 2 diabetes mellitus after laparoscopic gastric banding and bypass. Surgery for obesity and related diseases : official journal of the American Society for Bariatric Surgery. 2011;7(6):691-6.
68. Blackstone R, Bunt JC, Cortes MC, Sugerman HJ. Type 2 diabetes after gastric bypass: remission in five models using HbA1c, fasting blood glucose, and medication status. Surgery for obesity and related diseases : official journal of the American Society for Bariatric Surgery. 2012;8(5):548-55.
69. Schauer PR, Bhatt DL, Kirwan JP, Wolski K, Brethauer SA, Navaneethan SD, et al. Bariatric Surgery versus Intensive Medical Therapy for Diabetes — 3-Year Outcomes. New England Journal of Medicine. 2014;370(21):2002-13.
70. Hill NR, Levy JC, Matthews DR. Expansion of the homeostasis model assessment of β-cell function and insulin resistance to enable clinical trial outcome modeling through the interactive adjustment of physiology and treatment effects: iHOMA2. Diabetes care. 2013;36(8):2324-30.
71. Ali Aminian M, Sangeeta Kashyap M, David Cummings M, Deepa V Cherla M, Philip Schauer M, Roman Vangoitsenhoven MP. Presence Of Liver Steatosis Is Associated With Greater Diabetes Remission After Gastric Bypass Surgery. Obesity Week Conference 2019.
72. Steven S, Hollingsworth KG, Small PK, Woodcock SA, Pucci A, Aribisala B, et al. Weight Loss Decreases Excess Pancreatic Triacylglycerol Specifically in Type 2 Diabetes. Diabetes care. 2016;39(1):158-65.

AUTHOR CONTRIBUTIONS JSK, TT, SRB, AB, AA, SP and AP developed the protocol. JSK drafted the paper and TT, SRB, AB, AA, SP, KM and AP provided critical revisions.

FUNDING This work was supported by NIHR grant number [DRF-2017-10-042]. JSK is funded by a National Institute for Health Research (NIHR) Doctoral Research Fellowship

For peer review only - http://bmjopen.bmj.com/site/about/guidelines.xhtml

for this research project. The views expressed are those of the author(s) and not necessarily those of the NIHR or the Department of Health and Social Care.

CONFLICTS OF INTERESTS STATEMENT. None declared

ETHICS APPROVAL This study was approved by the London Bloomsbury Ethics Committee (REC reference number 18/LO/0931).

For peer review only

For peer review only - http://bmjopen.bmj.com/site/about/guidelines.xhtml
